# The Interplay Between Ca^2+^ Homeostasis, Endoplasmic Reticulum Stress, and the Unfolded Protein Response in Human Diseases

**DOI:** 10.3390/cells15040352

**Published:** 2026-02-15

**Authors:** Elia Ranzato, Simona Martinotti

**Affiliations:** DiSIT—Dipartimento di Scienze e Innovazione Tecnologica, University of Piemonte Orientale, Viale Teresa Michel 11, 15121 Alessandria, Italy; simona.martinotti@uniupo.it

**Keywords:** ER stress, unfolded protein response (UPR), calcium dyshomeostasis

## Abstract

**Highlights:**

**What are the main findings?**

- The Ca^2+^-ER Stress–UPR is a pivotal, highly sensitive signaling hub that connects Ca^2+^ homeostasis within the ER to protein quality control, cell fate, and a variety of downstream pathophysiological responses.
- The process is bidirectional with a self-amplifying mechanism, such that the result of Ca^2+^ depletion in the ER, whether attributed to SERCA malfunction, leak channels, or UPR, leads to suppression of PERK-CHOP, a maladaptive component of UPR, which in turn suppresses SERCA, thus entrapping the cell in a Ca^2+^-depleted, pro-apoptotic state.

**What are the implications of the main findings?**

- The Ca^2+^-ER Stress–UPR system is a common fundamental pathological mechanism that explains several diversified diseases, including neurodegeneration, CVD, and cancer, thus identifying a single, multi-disease target.
- The targeted therapy should be directed at the modulation of ER Ca^2+^ homeostasis, e.g., via activators of SERCA, or, alternatively, at the pharmacological redirection of the UPR from the maladaptive, CHOP-mediated phase to the adaptive, pro-survival phase.

**Abstract:**

The maintenance of endoplasmic reticulum (ER) Ca^2+^ homeostasis is intrinsically linked to the fidelity of protein folding, forming a functional tether that, when disrupted, triggers the Unfolded Protein Response (UPR). This bidirectional axis serves as a critical rheostat for cellular viability, yet its chronic dysregulation underpins the molecular etiology of numerous pathologies, including neurodegeneration, heart failure, and malignant transformation. This review provides a comprehensive interrogation of the Ca^2+^-ER Stress–UPR network, delineating how primary stress sensors—PERK, IRE1alpha, and ATF6—engage in complex feedback loops that either reinstate equilibrium or commit the cell to apoptosis. We specifically examine the PERK-CHOP-SERCA2b inhibitory circuit as a central driver of persistent Ca^2+^ depletion and discuss the role of Mitochondria-Associated Membranes (MAMs) in governing lethal Ca^2+^ transfer. Notably, we move beyond the classical paradigm of CHOP as a terminal apoptotic executioner, incorporating emerging evidence of its context-dependent adaptive functions. By synthesizing mechanistic insights across diverse disease models, this work highlights the transition from adaptive to maladaptive UPR as a universal pathological checkpoint. Ultimately, we evaluate the therapeutic potential of ‘axis-targeted’ interventions, such as SERCA activators and selective UPR modulators, aimed at resolving the underlying Ca^2+^ signaling defects in ER stress-related disorders.

## 1. Introduction

The ER is an active membrane network filling the cytoplasm of eukaryotic cells categorized structurally and functionally into the Rough ER, responsible for protein translation and the Smooth ER involved in lipid metabolism and detoxification [1]. Its vast surface area and unique internal environment enable the ER to perform its roles as the cellular site for protein production and quality assurance as well as the principal intracellular Ca2+ store. The operational interaction between these two functions is complex, involving elaborate processes that establish the ER as a crucial component in stress detection and signaling networks that regulate cellular homeostasis and fate [2].

### 1.1. Interconnected Role of Protein Folding and Ca^2+^ Signaling

#### 1.1.1. Protein Quality Control and ER Ca^2+^ Dependence

The lumen of the ER provides an ideal biochemical setting for the folding and post-translational modification of proteins. This is reliant on the presence of Ca^2+^-dependent chaperone proteins, such as BiP/Grp78 (binding immunoglobulin protein (BiP) also referred to as 78 kDa glucose-regulated protein) and the calnexin/calreticulin network [3].

The activity of these chaperones—responsible for folding and triaging terminally misfolded proteins for Endoplasmic Reticulum-Associated Degradation (ERAD)—is strongly sensitive to luminal Ca^2+^, which usually falls in the 100- to 500 μM range.

#### 1.1.2. The ER as the Principal Ca^2+^ Reservoir

The ER maintains a steep gradient of Ca^2+^ through the active functioning of Sarco/Endoplasmic Reticulum Ca^2+^-ATPase (SERCA) pumps [4]. The controlled, spatially and temporally precise discharge of this sequestered Ca^2+^ through Inositol trisphosphate receptors (IP_3_Rs) and ryanodine receptors (RyRs) produces intracellular cues, for coordinating metabolic activities regulating mitochondrial function through ER-mitochondria junctions or Mitochondria-Associated Membranes (MAMs) and managing gene expression. Any disruption in SERCA activity or Ca^2+^ channel operation directly impairs chaperone performance. Consequently, it triggers a decrease in protein quality regulation.

### 1.2. ER Stress and the Unfolded Protein Response Cascade

**Figure 1:**
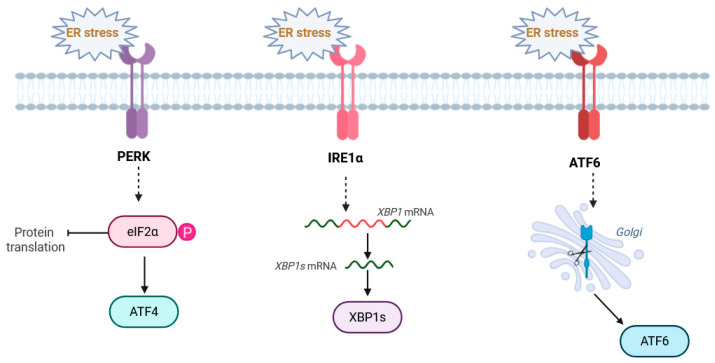
Schematic representation of UPR signaling pathways. The three primary UPR transducers—IRE1α, PERK, and ATF6—undergo activation upon the accumulation of misfolded proteins. This process is triggered when the chaperone GRP78 (BiP) is recruited to protein aggregates, resulting in its dissociation from the stress sensors. Created in BioRender. https://BioRender.com/0laj4yv (accessed on 25 January 2026).

ER stress is typically described as a condition where the ER is overwhelmed by the burden of produced proteins or exposed to harmful stress factors. This situation triggers the Unfolded Protein Response (UPR), a signaling system preserved across multicellular life forms, controlled by three sensors located in the ER [5] (see also Figure 1):

- PERK (PKR-like ER Kinase) inhibits global translation by phosphorylation of eIF2α but selectively enhances the transcription of the pro-survival factor ATF4 and the pro-apoptotic factor CHOP (C/EBP Homologous Protein).
- IRE1α, or inositol-requiring enzyme 1α, is an endoribonuclease responsible for the splicing of XBP1 mRNA into its active transcription factor form, XBP1s, which transcriptionally upregulates ERAD components and folding capacity.
- Activating Transcription Factor 6: ATF6 is cleaved in the Golgi to release an active domain that upregulates ER chaperones.

Although ER stress initially supports survival, prolonged or intense ER stress leads to stimulation of the PERK-ATF4-CHOP pathway, which eventually directs the cell towards apoptosis.

### 1.3. The Ca^2+^-ER Stress–UPR Axis in Human Pathophysiology

The tight functional interdependence within the Ca^2+^-ER Stress–UPR axis establishes it as a convergent pathological checkpoint across diverse disease spectrums. Rather than acting as isolated triggers, Ca^2+^ dyshomeostasis and protein misfolding form a self-amplifying cycle that dictates cellular fate in neurodegenerative, oncogenic, and cardiovascular contexts [6].

#### The Ca^2+^-UPR Axis in Major Human Pathologies

The central nervous system is exquisitely sensitive to ER perturbations, as neuronal signaling and synaptic plasticity rely on the rigorous compartmentalization of Ca^2+^. In disorders such as Alzheimer’s (AD) and Parkinson’s Disease (PD), the chronic accumulation of proteotoxic aggregates—specifically β-amyloid and α-synuclein—functions as a persistent stressor that destabilizes the ER environment.

Mechanistically, the chronic activation of PERK in neurons leads to the persistent phosphorylation of eIF2α, which causes a global translational shut-off of synaptic proteins. This is compounded by the CHOP-induced downregulation of SERCA2b, which depletes ER Ca^2+^ stores. The resulting Ca^2+^ dyshomeostasis triggers the activation of calpains, Ca^2+^-dependent proteases that further degrade the ER-mitochondria tethering proteins (such as MFN2), leading to mitochondrial fragmentation and an bioenergetic crisis that precedes neuronal death [7].

Clinical evidence suggests that this proteotoxicity is inextricably linked to primary Ca^2+^ defects; for instance, mutations in Presenilins (PS1/PS2) associated with familial AD do not merely alter proteolytic processing but directly impair SERCA pump efficiency and ER Ca^2+^ leak dynamics [4]. This chronic store depletion initiates a sustained, low-intensity UPR that eventually recruits the pro-apoptotic PERK-CHOP axis. The resulting synergy between mitochondrial Ca^2+^ overload, mediated by enhanced efflux through IP3R or Aβ pores, and chronic UPR signaling leads to the synaptic dysfunction and progressive neuronal loss characteristic of these pathologies [8]. In contrast to the degenerative loss of cells, malignancy often involves the co-option of the UPR to favor survival under the hostile conditions of the tumor microenvironment. Cancer cells frequently exhibit a “reprogrammed” Ca^2+^ metabolism that maintains the UPR in an adaptive phase, thereby avoiding the transition to apoptosis despite high biosynthetic demands, hypoxia, and nutrient deprivation [9].

The IRE1α-XBP1s and ATF4 pathways are typically hijacked to expand ER capacity and promote angiogenesis, while oncogenic adaptations often include the selective upregulation of SERCA isoforms or the modulation of IP3R expression to sustain proliferative signaling [10]. Intriguingly, the role of CHOP in this context appears increasingly complex; while traditionally viewed as a terminal executioner, emerging evidence suggests that transient or non-canonical CHOP signaling may, in specific metabolic landscapes, enhance tumor fitness and therapeutic resistance. Consequently, current pharmacological efforts aim to destabilize this precarious balance by using SERCA inhibitors to force a transition from adaptive survival to CHOP-mediated cell death [11].

In the tumor microenvironment, the IRE1α-XBP1s axis promotes survival by transcriptionally upregulating VEGF, thereby driving angiogenesis to counteract hypoxia. Furthermore, cancer cells often exploit the RIDD (Regulated IRE1-Dependent Decay) activity of IRE1α to selectively degrade mRNAs that encode pro-apoptotic factors, such as caspase-2. This molecular ‘shielding’ prevents the transition to the apoptotic UPR phase, even under conditions of severe proteotoxicity and nutrient deprivation [12].

In the cardiovascular system, where excitation–contraction coupling demands rapid and precise Ca^2+^ cycling, the ER (or sarcoplasmic reticulum, SR) Stress–UPR axis is a critical determinant of pathogenesis. In heart failure (HF), pathological phosphorylation of RyR channels by PKA or CaMKII induces a chronic “leakage” of Ca^2+^, which simultaneously impairs contractility and precipitates localized ER stress [13,14,15].

This stress triggers a maladaptive UPR that further depresses SERCA2a expression, creating a vicious cycle that drives myocardial fibrosis and contractile failure.

The initial trigger for UPR activation in HF is the chronic ER Ca^2+^ depletion caused by mechanical strain and neurohormonal overactivation. At the molecular level, this depletion induces the titration of BiP/GRP78 away from the luminal domains of PERK, IRE1α, and ATF6, as the chaperone is recruited to bind the accumulating misfolded proteins. Once activated, the PERK-ATF4 axis leads to the transcriptional downregulation not only of ATP2A2 (SERCA2a) but also of critical ion channel genes such as HCN4 (Hyperpolarization-Activated Cyclic Nucleotide-Gated channel 4) and KCND3 (potassium voltage-gated channel subfamily D member 3). This broader genomic reprogramming impairs both calcium reuptake and electrical stability, acting as a key driver of the transition to heart failure [16].

A similarly acute manifestation occurs during myocardial infarction; the sudden reperfusion following ischemia-induced energy depletion triggers a massive oxidative burst. This ROS surge inactivates SERCA and promotes exaggerated Ca^2+^ transfer to the mitochondria via Mitochondria-Associated Membranes (MAMs).

The resulting mitochondrial overload and subsequent opening of the permeability transition pore (mPTP) lead to irreversible cardiomyocyte apoptosis and tissue damage [17,18,19].

In pancreatic β-cells, chronic hyperglycemia induces an overwhelming demand for insulin synthesis, leading to localized ER stress. Mechanistically, this stress activates the PERK-ATF4-CHOP pathway, which increases the expression of TRB3 (Tribbles homolog 3). TRB3 acts as a negative regulator of Akt signaling, effectively blunting insulin sensitivity and promoting β-cell apoptosis. Simultaneously, the excessive Ca^2+^ efflux from the ER into the cytosol activates the NLRP3 inflammasome, linking ER stress directly to the chronic low-grade inflammation characteristic of Type 2 Diabetes [20].

## 2. The Ca^2+^-ER Stress–UPR Axis: An Intricate Molecular Link

The ER lumen is not a passive storage compartment; it is a chemically active environment where the two fundamental processes of protein folding and Ca^2+^ signaling are fully interrelated [21]. The disruption of one inevitably compromises the other, thus forming the core pathological axis that regulates cellular survival or apoptosis.

### 2.1. The Essential Role of Ca^2+^ in Maintaining ER Function

The folding of emerging polypeptides into their functional shapes is a highly coordinated process that relies significantly on a group of ER-resident chaperones, many of which are metalloproteins that need Ca^2+^, for peak functionality and structural stability [22].

The chaperones Calnexin (CNX) and Calreticulin (CRT) constitute elements of the ER quality control machinery facilitating the folding of N-linked glycoproteins. Their affinity for Ca^2+^ plays a role: a reduction in luminal Ca^2+^ levels significantly diminishes their ability to bind and release misfolded proteins effectively decelerating the folding process and causing protein buildup [23].

Serving as the controller of UPR and the most plentiful ER chaperone, the inherent ATPase function of BiP-GRP78 is allosterically influenced by Ca^2+^. A reduction in Ca^2+^ alters the shape and stability of BiP weakening its hold on newly formed proteins and UPR sensors (PERK, IRE1α, ATF6) thereby acting as an essential preliminary stage, in triggering UPR [24].

While their role in catalyzing disulfide bond formation largely relies on the redox state, the total folding efficiency facilitated by the Protein Disulfide Isomerases (PDIs) is optimized when ER Ca^2+^ homeostasis is well-maintained [19].

Therefore, the ER Ca^2+^ level functions as a rheostat establishing the folding capability of the whole ER network [25].

### 2.2. Molecular Causes and Consequences of ER Ca^2+^ Depletion

This essential ER Ca^2+^ gradient is preserved by an equilibrium between the absorption through the SERCA pumps and controlled discharge through the IP_3_R and RyR channels. The direct reason for Ca^2+^ depletion-triggered ER stress is abnormalities that disrupt this fragile balance [26].

#### 2.2.1. Dysfunction of SERCA Pumps

SERCA pumps are very sensitive to pathological insults [27], which results in impaired Ca^2+^ reuptake.

SERCA is abundant in reactive cysteine residues [28]. Elevated oxidative stress conditions, such as those seen in ischemia–reperfusion injury or chronic inflammation, cause S-nitrosylation or oxidation of these cysteine sites resulting in the loss of function and the pump’s failure to move Ca^2+^ against the electrochemical gradient [4].

Chronic ER stress and the subsequent activation of the UPR typically trigger a maladaptive signaling cascade, leading to the selective downregulation of SERCA2b transcription [29]. This repression is mediated either by the induction of the pro-apoptotic factor CHOP or through the action of specific microRNAs, such as miR-25 and miR-429, which target the SERCA2b transcript for post-transcriptional silencing [30]. Consequently, a self-perpetuating cycle of luminal Ca^2+^ depletion and persistent ER stress is established.

#### 2.2.2. Hyperactivity and Leakage of Ca^2+^ Release Channels

The major mechanism of depletion, especially in excitable cells, involves chronic leakage of Ca^2+^ [31].

Numerous disease-related signaling routes increase the responsiveness of Inositol trisphosphate receptors (IP_3_Rs) to IP_3_ concentrations causing unregulated Ca^2+^ puffing and sparking that exhausts the stores. IRE1α is also reported to degrade IP_3_R mRNAs through its RNase function though the resulting effects are intricate and vary depending on the context [32].

The molecular mechanism linking UPR to increased Ca^2+^ leak involves the induction of the pro-oxidant enzyme ERO1α via the PERK-CHOP pathway [33]. ERO1α-mediated oxidative stress leads to the S-nitrosylation of RyR2 cysteine residues. This post-translational modification causes the dissociation of the stabilizing protein FKBP12.6 (calstabin2) from the RyR2 complex, resulting in a ‘leaky’ channel. The subsequent diastolic Ca^2+^ leak into the cytosol creates a mitochondrial Ca^2+^ overload at the MAMs, which stimulates the electron transport chain to produce excessive ROS, thereby establishing a self-amplifying loop of oxidative damage and ER dysfunction.

The bridge between ER stress and ROS production lies at the MAMs. The Ca^2+^ leaked from the ER via oxidized RyR2 is directly funneled into the mitochondrial matrix through the Mitochondrial Calcium Uniporter (MCU). This mitochondrial Ca^2+^ overload overstimulates the Krebs cycle and the electron transport chain, leading to a burst of superoxide production. These mitochondrial ROS then diffuse back to the ER, further oxidizing RyR2 thiol groups and exacerbating the Ca^2+^ leak in a self-perpetuating loop [32].

### 2.3. The Molecular Interplay at MAMs: ER–Mitochondria Crosstalk

A critical locus for the Ca^2+^-ER Stress–UPR axis is provided by the Mitochondria-Associated Membranes (MAMs). These constitute micro-regions where the ER and mitochondria are physically connected forming confined zones, for rapid tightly localized Ca^2+^ exchange [34].

On MAMs, the IP_3_R channels on the ER membrane are in close proximity (∼10−80 nm) to the Voltage-Dependent Anion Channel on the outer mitochondrial membrane. This spatial coupling enables the high concentration of Ca^2+^ released from the ER to be taken up rapidly by the mitochondrial calcium uniporter [35]. This transport is crucial for activating TCA cycle enzymes and controlling ATP production.

Pathological ER stress is often correlated with a dramatic alteration in MAM structure and function [36]. Prolonged ER stress boosts the connection between ER and mitochondria (closer contact) causing excessive mitochondrial Ca^2+^ absorption. This Ca^2+^ surplus induces depolarization, elevated ROS generation and eventually activation of the intrinsic apoptotic pathway (release of cytochrome c). The ROS produced by the burdened mitochondria subsequently oxidizes SERCA and the lipids in the ER membrane forming a deadly feedback cycle of ER stress [37].

### 2.4. Adaptive vs. Apoptotic UPR: The CHOP Switch

Since the determination of fate is closely associated with the equilibrium between the adaptive and pro-apoptotic pathways of the UPR this signifies a shift that is greatly reliant on the intensity and duration of the Ca^2+^ depletion [38].

Mild, short-term ER stress promotes the immediate effects of UPR: global translational attenuation (PERK-eIF2α), increased folding capacity (ATF6), and enhanced degradation (IRE1α-XBP1s). These effects attempt to restore Ca^2+^ homeostasis indirectly by reducing protein load [39].

When ER stress persists, commonly indicated by Ca^2+^ depletion and issues, with MAMs the UPR shifts its role. Continuous PERK activation induces the production of CHOP or C/EBP Homologous Protein, known as the effector molecule of the UPR. CHOP facilitates apoptosis by increasing the pro-apoptotic Bcl−2 family proteins (Bim) and decreasing the anti-apoptotic proteins (e.g., Bcl−2) [40].

However, the traditional view of CHOP as a terminal pro-apoptotic executioner has been recently challenged by evidence suggesting a more nuanced, context-dependent role. While chronic induction remains a hallmark of cell death, transient or moderate CHOP expression might contribute to adaptive responses by modulating metabolic genes or facilitating the autophagy-mediated clearance of protein aggregates. This ‘dual-role’ hypothesis suggests that the biological output of CHOP signaling is not binary but is rather fine-tuned by its temporal dynamics and the specific stress landscape of the cell [41].

Additionally, CHOP reduces SERCA2b expression intensifying ER stress and thus reinforcing the condition of Ca^2+^ deficiency [4].

### 2.5. ER Stress and Ca^2+^ Signaling in Disease

The delicate Ca^2+^-dependent nature of neuronal and cardiac functions renders these tissues exquisitely sensitive to ER homeostatic perturbations [42].

In neurodegenerative disorders such as AD and PD, the chronic accumulation of misfolded proteins does not merely trigger the UPR but exploits the aforementioned PERK-CHOP-SERCA inhibitory loop to lock neurons in a state of persistent Ca^2+^ depletion [43].

For instance, mutations in Presenilins linked to familial AD impair ER Ca^2+^ leak, causing a low-level but constitutive UPR activation that eventually collapses the organelle’s folding capacity [44].

Similarly, in heart failure, the pathological phosphorylation of RyR leads to a ‘leaky’ state; this chronic Ca^2+^ efflux induces localized ER stress, which, via CHOP-mediated repression of SERCA2a, compromises contractile recovery and facilitates the transition to failure [45,46].

Conversely, in the context of malignancy, tumor cells often bypass the apoptotic threshold of this axis. By upregulating specific SERCA isoforms or modulating MAM-tethering proteins, cancer cells harness the adaptive branches of the UPR (IRE1alpha-XBP1s) to support high secretory demands and promote angiogenesis [47].

Understanding these tissue-specific exploitations of the Ca^2+^-UPR axis is crucial for developing targeted interventions that can either reinstate homeostasis in degenerating cells or force an apoptotic switch in resistant tumors [48,49].

## 3. Feedback Signaling and Crosstalk: UPR Regulation of Ca^2+^ Homeostasis

The UPR does not function as a one-way signaling route triggered by Ca^2+^ depletion; instead, it swiftly forms intricate feedback circuits aiming to recover ER Ca^2+^ concentrations or, if this fails, deliberately intensifies the depletion to induce cell death. The two-way interaction between UPR sensors and Ca^2+^ regulation systems is essential for determining cell destiny.

### 3.1. IRE1α: Modulating ER Biogenesis and Ca^2+^ Channels

The IRE1α branch, through the active transcription factor XBP1s, exerts powerful control over ER Ca^2+^ homeostasis.

XBP1s is a powerful transcriptional driver of genes involved in ER membrane synthesis, including components of lipid metabolism [50]. XBP1s acts as a transcriptional regulator for genes linked to ER membrane production encompassing elements of lipid metabolism [50]. By enlarging the ER size XBP1s boosts the protein folding capacity, notably elevating the production of SERCA isoforms (SERCA2b) that facilitate Ca^2+^ reabsorption. This serves as an adaptive response meant to directly mitigate Ca^2+^ loss [50].

Regulated beyond splicing XBP1 mRNA, the IRE1α endoribonuclease activity can cleave and degrade other mRNAs, a process termed Regulated IRE1-Dependent Decay (RIDD) [51]. Among the RIDD targets are mRNAs encoding ER membrane proteins, including some SERCA regulators and potentially components of the IP_3_R channels [52]. The breakdown of proteins that regulate SERCA would effectively block Ca^2+^ reuptake thereby allowing the UPR to shift from a phase of adaptation to a pro-apoptotic pathway by hindering Ca^2+^ restoration.

### 3.2. PERK: The SERCA Repressor and MAM Mediator

The PERK pathway offers the molecular connection between UPR activation and the ultimate disruption of Ca^2+^ balance [53].

Sustained activation of PERK drives the expression of the pro-apoptotic factor CHOP [54]. CHOP directly acts as a negative regulator of the SERCA2b promoter, thereby repressing the transcription and synthesis of SERCA. This transcriptional repression decreases the cellular capability to pump Ca^2+^ into the ER and solidifies its depleted state, ensuring the continuance of the ER stress condition, which is a required step for UPR-induced apoptosis.

PERK activation also plays a role in regulating physical tethering between the ER and mitochondria at the MAMs [55]. Persistent PERK signaling enhances the development and maintenance of MAMs resulting in prolonged mitochondrial Ca^2+^ absorption. This overload of Ca^2+^ is extremely harmful and triggers Mitochondrial Outer Membrane Permeabilization (MOMP) along with the release of pro-apoptotic molecules such as cytochrome c [56].

### 3.3. The ATF6 Branch and Ca^2+^-Dependent Refolding

The ATF6 pathway primarily serves as a mechanism designed to specifically alleviate the stress triggered by Ca^2+^ depletion.

The functional N-terminal segment of ATF6 moves into the nucleus to trigger the expression of ER chaperones, such as BiP and GRP94. This surge of Ca^2+^-dependent folding assistants aims to boost the ER’s ability to manage misfolded proteins despite reduced Ca^2+^ levels, simultaneously allowing time for SERCA function to be restored.

### 3.4. Ca^2+^ Signaling as a Regulator of UPR Output

One may note that the crosstalk is not unidirectional. Altered ER Ca^2+^ levels may also, in a manner independent of BiP dissociation, directly modulate both the activity and output of UPR sensors.

STIM1, the main ER Ca^2+^ sensor that regulates Store-Operated Calcium Entry (SOCE), physically interacts with the IRE1α luminal domain [57]. Upon ER Ca^2+^ depletion, the clustering of STIM1 not only triggers SOCE but also activates and induces the clustering of IRE1α, indicating that Ca^2+^ signaling directly regulates the IRE1α dimerization and RNase activity [58].

Low Ca^2+^ generally activates the UPR, but various reports suggest that the IRE1α RNase domain requires specific local Ca^2+^ concentrations for its maximum activity, indicating that the precise spatial and temporal dynamics of Ca^2+^ release can tune the specific output of the UPR branches, favoring, for example, XBP1 splicing over RIDD [59].

**Figure 2:**
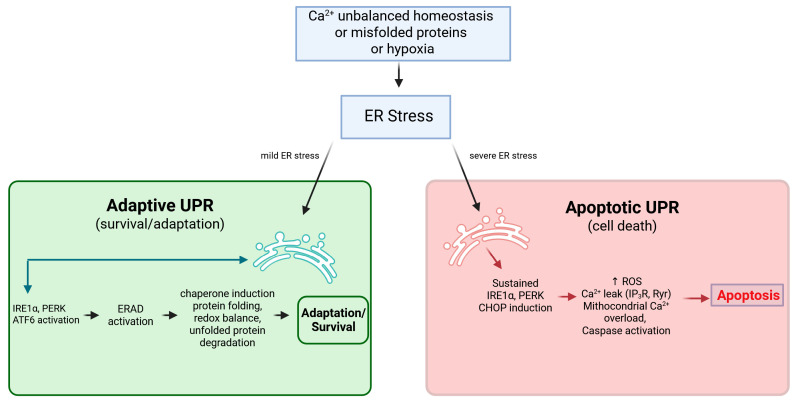
Molecular mechanisms of the adaptive versus apoptotic Ca^2+^-ER Stress–UPR interplay. Under physiological conditions or mild stress (green pathway, at the left), the activation of UPR sensors leads to the transcription of chaperones (e.g., BiP/GRP78) and foldases, aiming to restore protein folding capacity and ER Ca^2+^ homeostasis. Conversely, under chronic or unresolved stress (red pathway, at the right), the signaling shifts toward a terminal UPR program. This involves the sustained activation of PERK and the upregulation of CHOP, which promotes ER Ca^2+^ leakage (e.g., via IP_3_R stabilization), leading to mitochondrial Ca^2+^ overload and the triggering of the apoptotic cascade. Created in BioRender. https://BioRender.com/d3mjswb (accessed on 12 February 2026).

In summary, the UPR is a high-powered signal processor that, in response to Ca^2+^-mediated stress, strives to reinstate homeostasis but contains robust negative feedback mechanisms, such as the CHOP-SERCA loop, that lock the cell into a pathological, Ca^2+^-depleted, pro-apoptotic state if the stress is unresolved [41] (see also Figure 2).

### 3.5. Pathological Implications of UPR-Mediated Ca^2+^ Dysregulation

The key shift from recovery to pro-apoptotic cell death is often determined by whether the UPR can successfully reestablish ER homeostasis. In significant human diseases the harmful pathway often forms when the UPR positive feedback loops (such as CHOP inhibiting SERCA) remain persistently active, surpassing the original adaptive reactions.

In advancing diseases like AD and PD the continuous existence of misfolded protein accumulations (β-amyloid, α-synuclein) causes sustained mild ER stress. Neurons experiencing this stress frequently show ongoing activation of the PERK signaling pathway. The induced production of CHOP creates a self-sustaining loop of injury: CHOP inhibits the transcription of SERCA2b leading to a lasting reduction, in the neuron’s capacity to replenish its ER Ca^2+^ reservoirs [60].

This Ca^2+^ depletion inhibits ER chaperones guaranteeing continued protein misfolding while also preparing the neuron for apoptosis. The inability of the UPR to restore Ca^2+^ balance is regarded as a mechanism underlying the ongoing neuronal degeneration typical of these disorders.

In multiplying cancer cells, the adaptive arm of the UPR is frequently hijacked to promote unchecked proliferation, involving the adjustment of Ca^2+^ regulation to aid survival within the challenging tumor microenvironment.

The IRE1alpha/XBP1s axis is frequently hyperactivated in highly aggressive tumors, such as multiple myeloma and triple-negative breast cancer [61]. In addition to enhancing protein folding ability XBP1s promotes the expression of SERCA and lipid synthesis genes aiding the cancer cell in enlarging its ER volume and preserving membrane stability essential, for meeting the biosynthetic needs of rapid proliferation.

This XBP1s-driven maintenance of ER capacity allows the cancer cell to buffer the Ca^2+^ signaling necessary for cell cycle progression while avoiding the switch to CHOP-mediated apoptosis. Targeting IRE1alpha/XBP1s has become a therapeutic strategy aimed at destabilizing ER Ca^2+^ balance and pushing the tumor cell towards death.

In conditions of severe metabolic compromise like myocardial ischemia, the tight regulatory control of the UPR over Ca^2+^ flux at the MAMs becomes highly pathogenic [62].

Acute stress, such as ischemia–reperfusion injury, strongly activates the PERK pathway [63]. Persistent PERK signaling has been shown to increase the physical tethering and stability of the MAMs, leading to an exaggerated proximity between ER IP_3_R and mitochondrial Ca^2+^ uptake machinery [64].

This enhanced ER-to-mitochondria coupling, driven by UPR signaling, results in a fatal mitochondrial Ca^2+^ overload. This overload directly triggers MOMP and the release of cytochrome c, causing massive cardiomyocyte apoptosis and contributing significantly to the irreversible damage following a heart attack [65]. The UPR’s attempt to compartmentalize and manage stress via the MAMs thus ironically serves as a conduit for cell death.

## 4. Targeting the Ca^2+^-ER Stress–UPR Axis: Therapeutic Implications

Given the disease-causing involvement of the Ca^2+^-ER Stress–UPR pathway across a broad range of conditions including neurodegenerative disorders, cardiovascular diseases and cancer, this signaling route has emerged as an attractive target for new treatment approaches [66,67]. The goal would be to redirect the UPR from its harmful/apoptotic stage back to its protective/pro-survival phase or to reestablish normal ER Ca^2+^ balance. Re-establishing the balance between Ca^2+^ uptake and release is the most direct means of relieving ER stress [68]. Small-molecule enhancers designed to boost the pump function of SERCA are being developed, such as those aimed at SERCA2a in heart failure. By enhancing Ca^2+^ uptake these agents replenish the Ca^2+^ reservoir thereby reactivating Ca^2+^-dependent chaperones and ultimately addressing the ER stress signal at its origin [69].

Because excessive MAM tethering promotes lethal mitochondrial Ca^2+^ overload, the disruption of these physical tethers—for example, by targeting the protein complexes such as Miro/Mitofusin2 that mediate the ER—mitochondria contact—can selectively block the pro-apoptotic ROS-amplification loop without globally affecting either ER or mitochondrial function [70].

Inhibitors targeting the IRE1α RNase domain block splicing of XBP1 and RIDD is an approach particularly relevant in malignancies where the product of XBP1s is actively hijacked to support tumor survival (e.g., multiple myeloma). By blocking XBP1s, these agents aim to strip the tumor cells of their adaptive survival mechanism [71].

In certain settings, PERK inhibitors must be used together with XBP1s inhibitors to completely suppress the UPR. A more subtle approach is to enhance the adaptive side while suppressing the maladaptive side; for example, activating ATF6 while inhibiting PERK [72].

The Ca^2+^-ER Stress–UPR axis offers various points of intervention, beyond general anti-apoptotic or anti-inflammatory drugs. The basis of highly selective therapies will come via understanding the molecular feedback loops that display either a positive or negative influence of Ca^2+^ on UPR and vice versa. Future treatments for neurodegeneration, cardiovascular failure, and certain cancers may very well rely on molecules that, through pharmacological means, restore ER proteostasis [73,74] by either directly correcting the Ca^2+^ balance or shifting UPR back toward an adaptive, sustainable response.

## 5. Conclusions

The evidence synthesized in this review establishes the Ca^2+^-ER Stress–UPR axis as a critical and bidirectional signaling node. The fundamental conclusion is that the crosstalk between ER Ca^2+^ dynamics and the UPR is self-amplifying: while Ca^2+^ depletion acts as a primary trigger for stress, the subsequent maladaptive UPR reinforces this depletion through transcriptional and post-translational feedback loops.

This axis represents a convergent pathological hallmark across neurodegeneration, cardiovascular diseases, and cancer. The transition from an adaptive to a pro-apoptotic UPR serves as a universal checkpoint, often governed by the duration of Ca^2+^ dyshomeostasis and the stability of ER-mitochondria contacts at the MAMs. Consequently, pharmacological efforts should move beyond broad UPR inhibition. Future therapeutic success likely resides in the development of ‘rheostat-modulators’—such as SERCA activators or selective CHOP-pathway inhibitors—capable of diverting the UPR from its terminal apoptotic phase toward a sustainable, homeostatic response. Resolving the root Ca^2+^ signaling defect, rather than merely mitigating downstream symptoms, remains the most promising frontier for treating ER stress-related pathologies.

## Data Availability

No new data were created or analyzed in this study.
